# Systemic lupus erythematosus and epilepsy: A Mendelian randomization study

**DOI:** 10.1002/epi4.13058

**Published:** 2024-09-28

**Authors:** Yang Hu, Duo Lin, Dongmei Wu, Yuqing Zhang, Gongbo Li

**Affiliations:** ^1^ Department of Neurology The Second Affiliated Hospital of Chongqing Medical University Chongqing China; ^2^ Department of Neurology Zhongshan Hospital of Traditional Chinese Medicine Guangdong China

**Keywords:** epilepsy, Mendelian randomization, systemic lupus erythematosus

## Abstract

**Objective:**

Numerous observational studies have found a relationship between systemic lupus erythematosus (SLE) and epilepsy; however, their causal relationship remains unclear. This study aimed to investigate the causal role of SLE in epilepsy or any of its subtypes using a two‐sample Mendelian randomization (MR) analysis.

**Methods:**

Single nucleotide polymorphisms (SNPs) linked to SLE were utilized as instrumental variables in MR analysis to assess their causal impact on epilepsy. The primary MR findings were derived using the inverse variance weighted (IVW) method, which was further supported by the weighted median and MR‐Egger regression techniques. Additionally, sensitivity analyses, including Cochran's Q test and pleiotropy tests, were conducted to evaluate the influence of these SNPs on epilepsy, particularly looking for signs of horizontal pleiotropy and heterogeneity.

**Results:**

We selected 43 SNPs that reached genome‐wide significance from genome‐wide association studies (GWASs) on SLE to serve as instrumental variables in this study. The IVW method showed no evidence to support a causal association between SLE and epilepsy (all epilepsy: odds ratio (OR) = 1.006, 95% confidence interval (CI) = 0.994–1.018; focal epilepsy: OR = 1.006, 95% CI = 0.994–1.019; generalized epilepsy: OR = 1.015, 95% CI = 0.996–1.035). Other MR complementary methods revealed consistent results. Furthermore, there was no evidence indicating heterogeneity or horizontal pleiotropy.

**Significance:**

The findings of MR analysis did not support a genetically predicted causal relationship between SLE and epilepsy, but emphasized the need for further research on shared pathophysiological mechanisms, particularly the role of immune system abnormalities and potential influences such as chronic inflammation and therapeutic interventions.

**Plain Language Summary:**

The etiology of epilepsy is complex and diverse, including immune factors. Through a Mendelian randomization analysis, we did not find evidence of a genetic causal relationship between systemic lupus erythematosus and epilepsy. However, this does not invalidate epidemiological evidence, and further exploration is needed to investigate factors influencing the relationship between the two.

**Key points**

- Mendelian randomization (MR) was used to investigate the causal role of systemic lupus erythematosus (SLE) in epilepsy or any of its subtypes.
- The two‐sample MR analysis did not support a genetically predicted causal relationship between SLE and epilepsy.

## 1 INTRODUCTION

Systemic lupus erythematosus (SLE) is a chronic, systemic autoimmune disease that causes damage to multiple organ systems.^1^ Clinically, SLE can affect numerous cells, tissues, and organs, and heterogeneity in both severity and the target organ affected is common. Manifestations of SLE may include inflammation and damage to the skin, joints, kidneys, central nervous, and cardiovascular systems. Significantly, SLE predominantly affects women, occurring nine times more frequently than in men. Furthermore, SLE ranks among the top 20 causes of death for women aged 5–64 years in the United States, particularly impacting women of African or Hispanic descent,^2^ thus highlighting its significance as a public health concern.

Epilepsy is a common neurological disease characterized by sudden abnormal discharge of the brain, resulting in transient brain dysfunction.^3^ The etiology of epilepsy is multifaceted, with the immune system and related immunological factors playing a crucial role. Observational studies and meta‐analyses have consistently shown that the incidence of epilepsy is higher among patients with systemic lupus erythematosus.^4^, ^5^, ^6^ A cross‐sectional study compared SLE patients with an age and gender‐matched control group to understand the incidence of epilepsy, revealing a significantly higher proportion of epilepsy in SLE patients (4.03% vs. 0.87%, *p* < 0.001).^7^ The results of a cohort study showed that the incidence of epilepsy was 2.86‐fold higher in the SLE cohort than in the non‐SLE cohort (9.10 per 10 000 person‐years vs. 3.18 per 10 000 person‐years). Nevertheless, the inherent limitations of observational studies, including the risks of reverse causation and residual confounding, constrain the clarity of SLE's impact on epilepsy.

Mendelian randomization (MR) is a method that uses genetic variations as instrumental variables to assess whether observed associations are causal. In two‐sample MR, causal effects are estimated using data from different samples: one related to the exposure and another to the outcome. To our knowledge, no research has yet utilized the MR method to investigate the causal relationship between the risks of SLE and epilepsy. Therefore, this study's principal objective is to confirm the existence of a causal link between SLE and epilepsy through a comprehensive two‐sample MR analysis.

## 2 METHODS

### 2.1 Data sources and selection of genetic variants

Genetic associations of SLE were retrieved from the largest public genome‐wide association study (GWAS) meta‐analysis, which included 7219 cases and 15 991 controls.^8^ In this dataset, the study found that 15 984 SNPs with strong associations (*p* < 5 × 10^−8^) with SLE had been screened out from the SLE GWAS data.^9^ Following the clumping process, 43 SNPs exhibiting no linkage disequilibrium (LD) were carefully chosen. Importantly, none of the minor allele frequencies (MAFs) were less than 0.01. We accessed the relevant open‐access data from this article.

Furthermore, we obtained comprehensive GWAS statistical data on epilepsy‐related subtypes from the International League Against Epilepsy (ILAE) data in the IEU Open‐GWAS project database, which brought together genome‐wide data on a total of 15 212 epilepsy cases and 29 677 controls. As for epilepsy subtypes, summary statistics for focal epilepsy (9671 cases) and generalized epilepsy (3769 cases) were also obtained. Table 1 displays the sample sizes of the datasets. More detailed information on epilepsy cases can be found in the original study.^10^

**TABLE 1:** The characteristics of our study included GWAS dataset for SLE and epilepsy.

Trait	Consortium	Sample size	Population (%European)
SLE	‐	23 210 (case: 7219, control: 15991)	100
Epilepsy	ILAE		
All epilepsy		44 889 (case: 15212, control: 29677)	95.5
Focal epilepsy		39 348 (case: 9671, control: 29677)	94.0
Generalized epilepsy		33 446 (case: 3769, control: 29677)	98.3

### 2.2 Statistical analysis for Mendelian randomization

Three methods of MR were used in this study to analyze the causal effect of SLE on epilepsy, including the IVW method, the MR‐Egger regression method, and the weighted median method. Each of these methods employs a unique statistical model to assess causal relationships, which, in turn, validates the robustness of the findings. The IVW is considered the primary method of analysis as it is the gold standard for MR inference. It is primarily used for basic causal estimation and provides accurate results by calculating a weighted average of the Wald ratio estimates.^11^ On the other hand, the MR‐Egger method is used to detect sensitivities and it provides calculations after adjusting for pleiotropy.^12^ Lastly, the median‐weighted method, which allows for the presence of a 50% null of SNPs, is applied to ascertain the causal estimates.^13^ Phenoscanner was used to find SNPs associated with outcomes.

All analyses were conducted by using the “TwoSampleMR” package in R software (Version 4.1.3).

### 2.3 Heterogeneity and sensitivity test

We evaluated the heterogeneities between SNPs using Cochran's Q‐statistics and conducted a pleiotropy test to examine the polymorphism.^12^, ^14^ Additionally, we performed a “leave‐one‐out” analysis to determine whether the observed causal association could be attributed to a single SNP.

## 3 RESULTS

Basic information of the contributing GWAS is summarized in Table 1.

### 3.1 Effects of SLE on epilepsies

**FIGURE 1:**
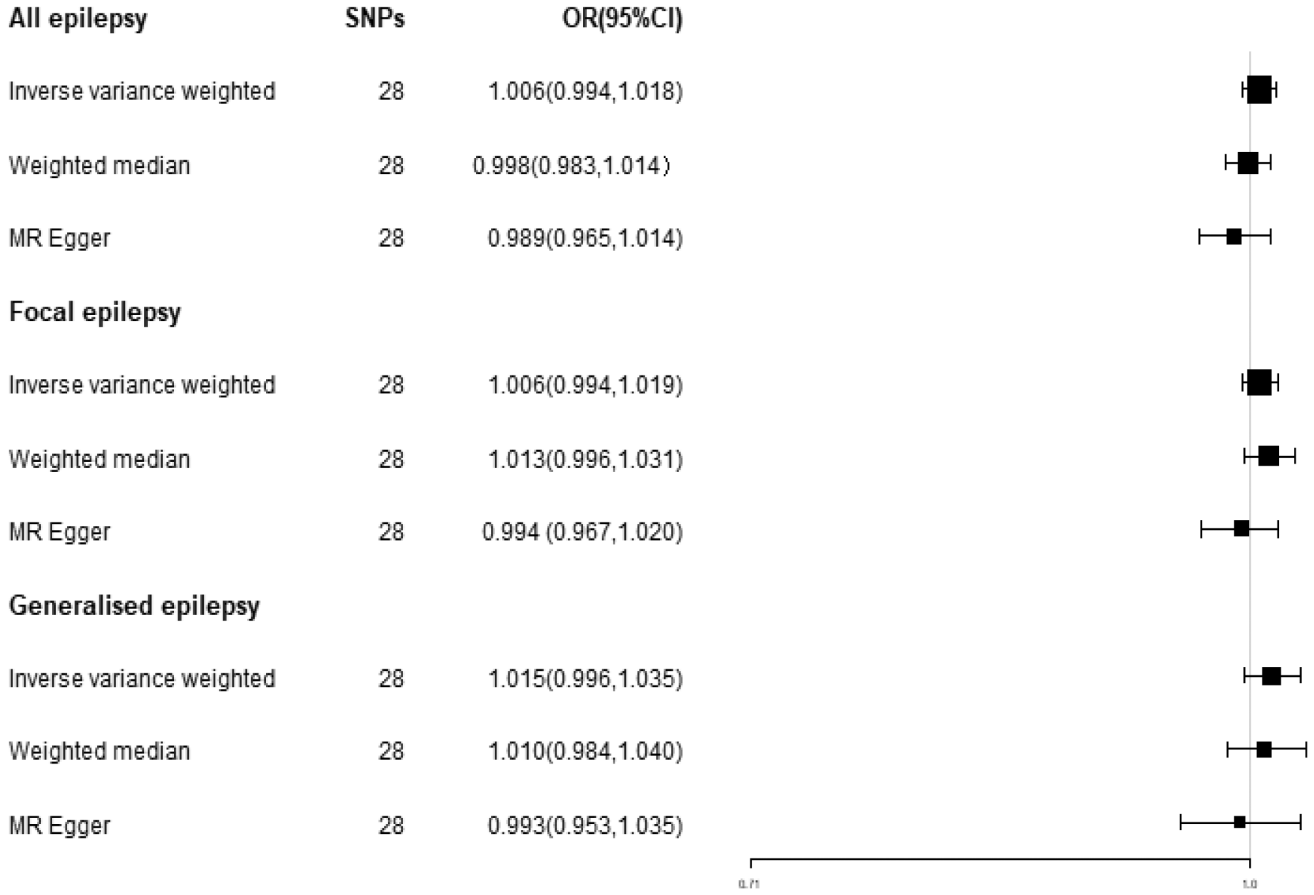
Forest plot of MR estimates of the causal effect of SLE on epilepsy.

The results of the IVW test method indicated that there was no observed effect of SLE on any form of epilepsy, including both overall epilepsy (OR = 1.006, 95% CI = 0.994–1.018, *p* > 0.05) and focal epilepsy (OR = 1.006, 95% CI = 0.994–1.019, *p* > 0.05). Similarly, the analysis of generalized epilepsy yielded an OR of 1.015, with a 95% CI of 0.996–1.035, further indicating a non‐significant effect (*p* > 0.05) (Figure 1). These findings were consistent across the weighted median method and MR‐Egger regression results.

### 3.2 Pleiotropy and sensitivity analysis

**FIGURE 2:**
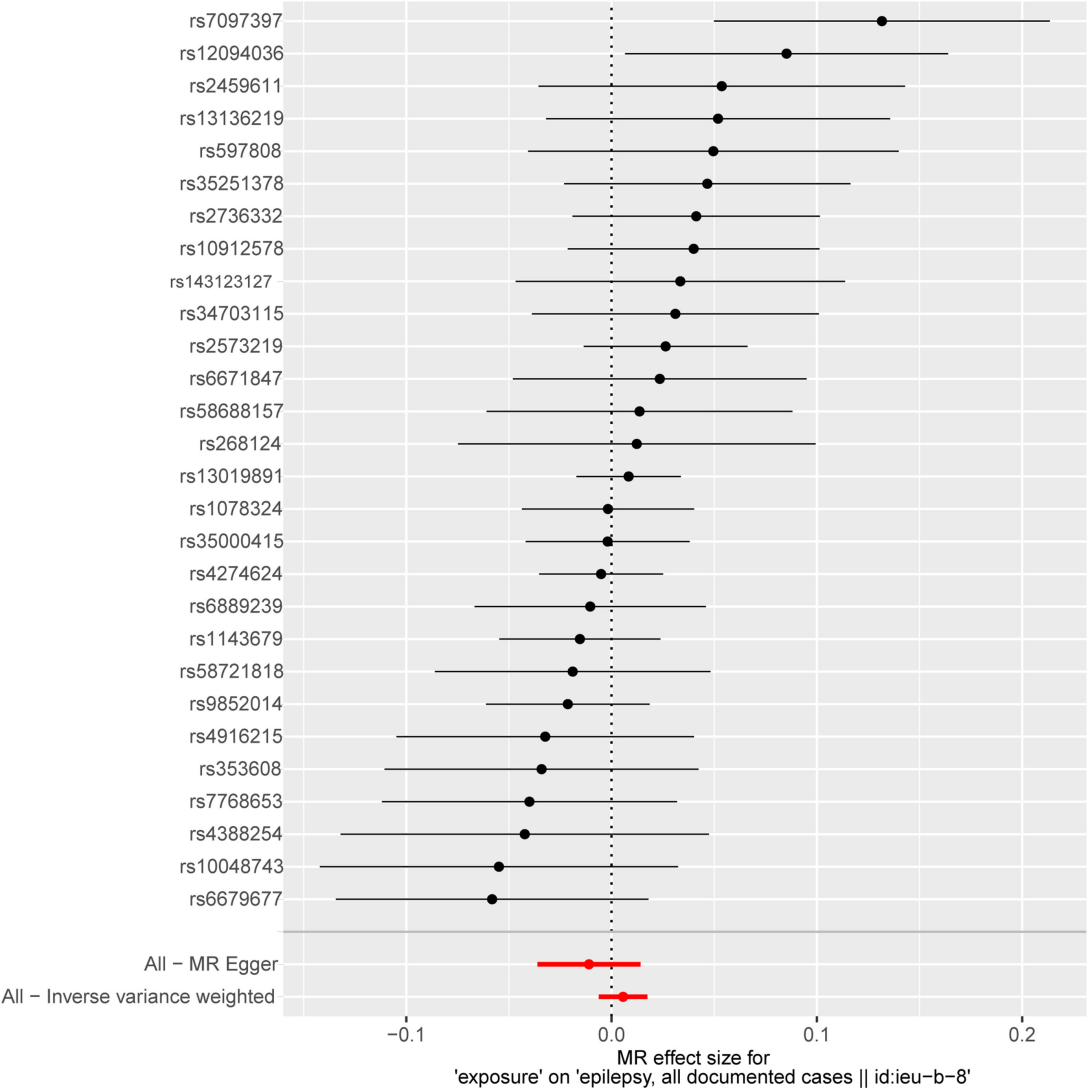
Forest plot of focal epilepsy for each one SD increase of epilepsy risk.

**FIGURE 3:**
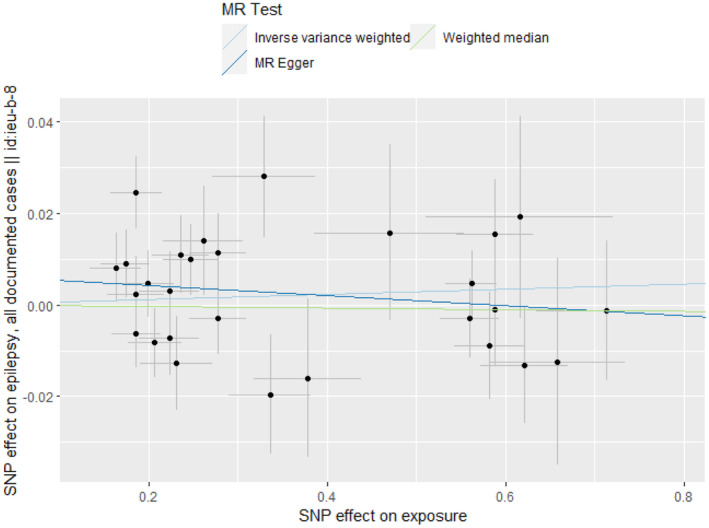
Scatter plot showing the effect of genetic instruments on epilepsy risk against their effect on all epilepsy.

**FIGURE 4:**
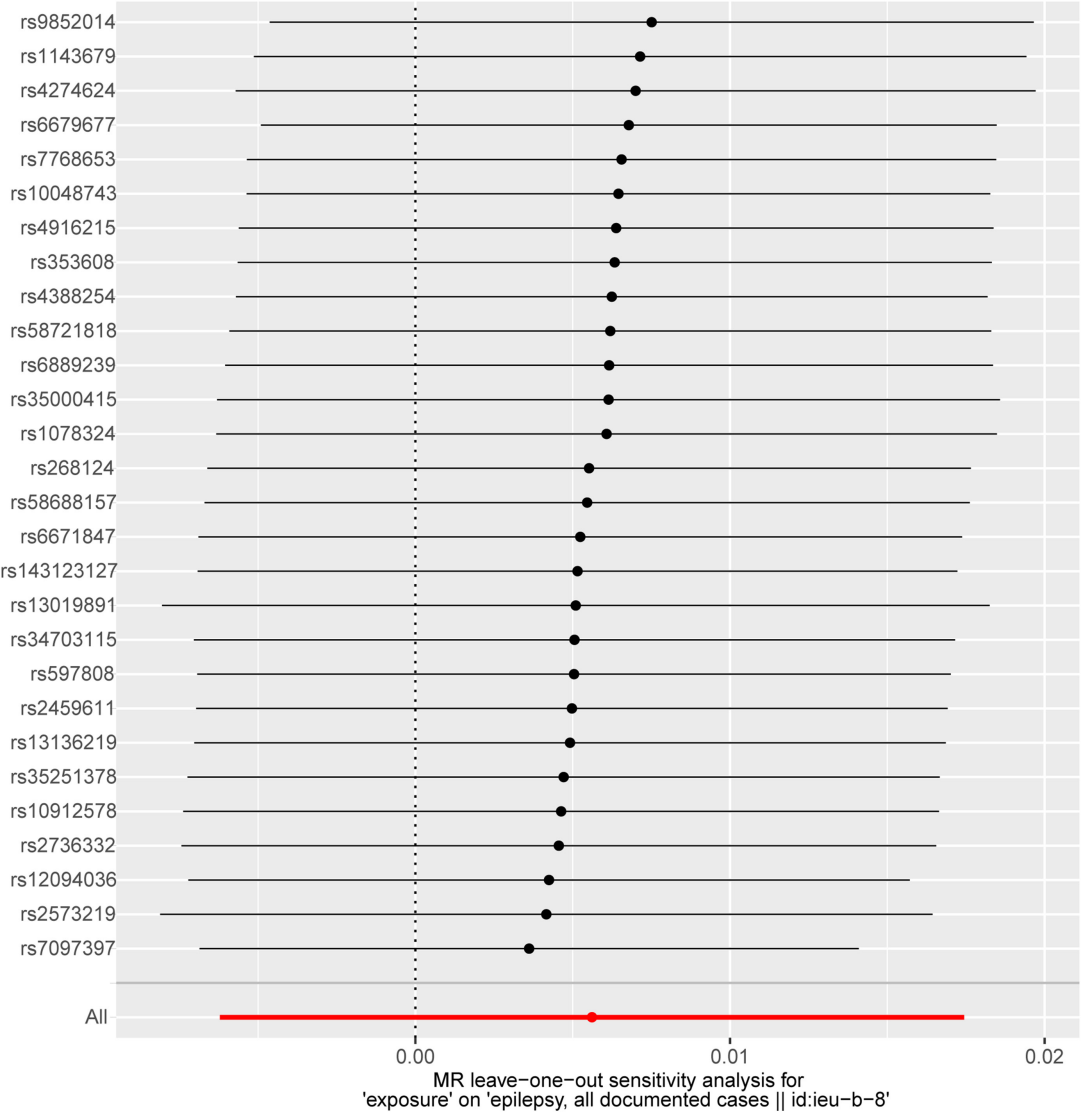
There was no substantial change in the IVW causal estimate after removing any of the instrumental SNPs.

**FIGURE 5:**
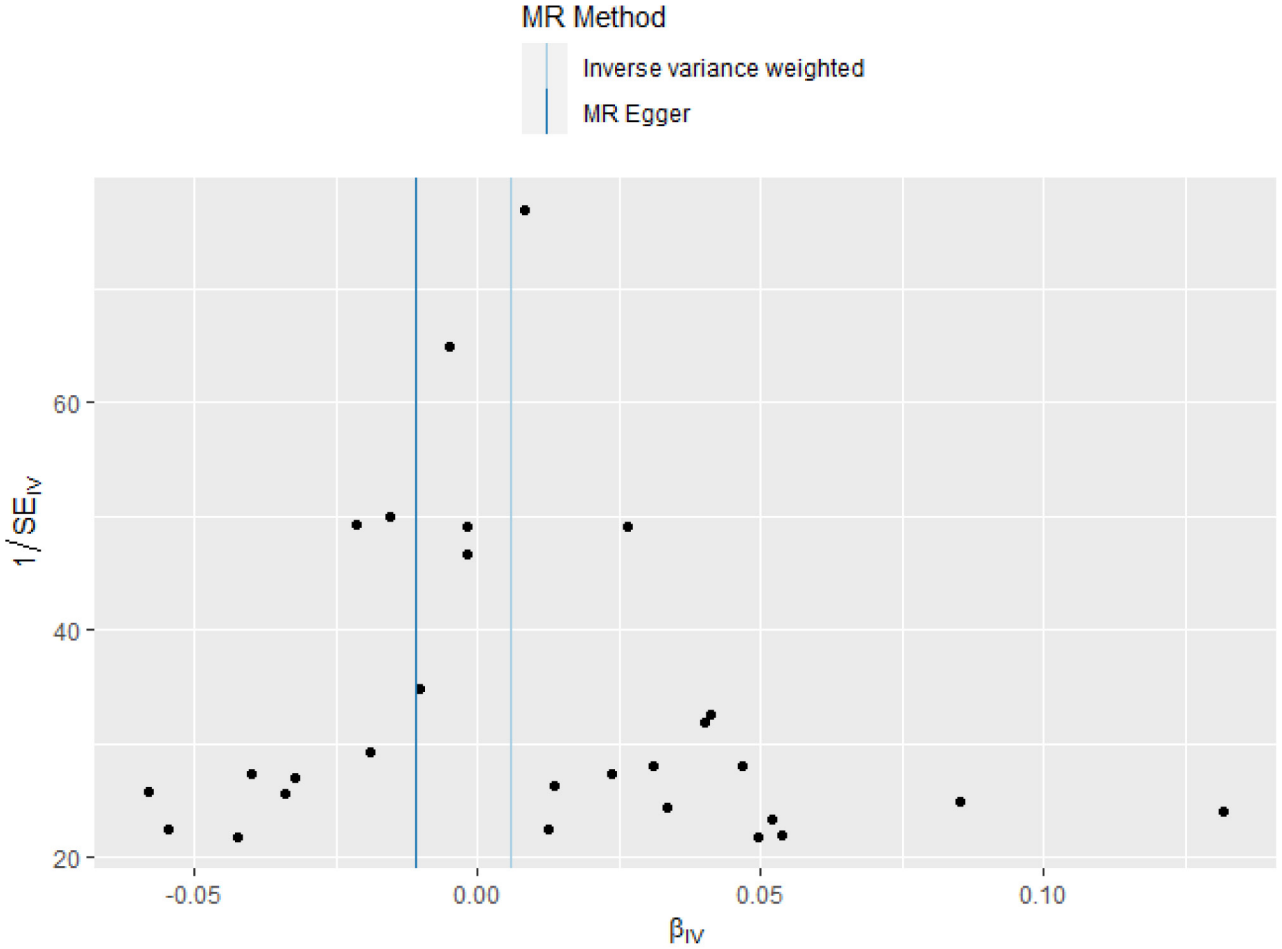
The funnel plot showed no asymmetry.

The heterogeneity test did not find any heterogeneity among the selected IVs. MR‐Egger regression and the MR‐PRESSO global test showed no horizontal pleiotropy between the IVs and outcomes (Table 2). To further this investigation, we selected 28 independent SNPs from GWAS on SLE as the IVs, all of which have shown a strong association with SLE at genome‐wide significance. However, despite these associations, the IVW method and MR‐Egger analysis indicated no causal association between SLE and all epilepsy, with *p*‐values greater than 0.05 (Figures 1 and 2). To visually represent these findings, scatter plots are presented in Figure 3, elucidating the primary outcomes. Moreover, the leave‐one‐out analysis reinforced the robustness of these results, suggesting that no single instrumental SNP drives the causal effect (Figure 4). Lastly, the funnel plot affirmed the validity of our findings by showing no evidence of asymmetry, thereby suggesting a low risk of directional pleiotropy (Figure 5). We found similar results in focal epilepsy and generalized epilepsy (Figures S1 and S2).

**TABLE 2:** Evaluation of heterogeneity and pleiotropy using different methods.

Exposure	Outcome	Pleiotropy test			Heterogeneity test			
		MR‐Egger			MR‐Egger		IVW	
		Intercept	SE	*p*	*Q*	*Q*‐pval	*Q*	*Q*‐pval
SLE	All epilepsy	−0.006	0.004	0.155	33.433	0.149	36.182	0.111
	Focal epilepsy	0.004	0.004	0.320	28.205	0.348	29.319	0.345
	Generalized epilepsy	0.008	0.007	0.246	30.517	0.246	32.168	0.225

Abbreviations: Q_pval, the *p*‐value of Cochran's *Q* test; *SE*, Standard error of Beta.

## 4 DISCUSSION

Immune system diseases have always been considered a risk factor for the occurrence of epilepsy.^15^ However, whether there is a specific causal relationship between SLE and epilepsy is still uncertain. We used three different estimation methods to conduct MR analysis. There is no statistically significant causal association between genetically predicted SLE and the risk of epilepsy. These results were corroborated by multiple sensitivity analyses.

Many surveys have demonstrated the emergence of epilepsy and seizures subsequent to SLE. It has been reported that the prevalence of epilepsy among SLE patients is 6–27%.^16^, ^17^, ^18^ In certain case series, the rate goes up to 31.1–58%.^19^, ^20^ A study included 2203 patients with no history of epilepsy prior to diagnosis of SLE, finding that 157 (7.13%) experienced epileptic seizures.^21^ Another study recruited 519 SLE patients. The rate of epileptic seizures was 11.6% (88.3% of cases were acute seizures, while the remaining 11.7% were recurrent seizures). The determining factors for epileptic seizures were stroke, antiphospholipid antibodies, and nephritis.^22^ The Systemic Lupus International Collaborating Clinics (SLICC) performed a prospective inception cohort study. The research examined 1631 patients with SLE, with 4.6% experiencing epileptic seizures. The determining factors for the incidence were African race/ethnicity (risk ratio of 1.97), lower levels of education (risk ratio of 1.97), and disease activity.^23^ To our knowledge, most studies have only observed SLE patients without employing a control group of non‐SLE patients for risk comparison of epilepsy. A retrospective study design in Taiwan included 32 301 SLE patients and matched them with 129 204 control subjects, finding that the incidence of epilepsy in the SLE cohort was 2.86 times higher than in the non‐SLE cohort (9.10 vs. 3.18 cases per 10 000 person‐years). The complications significantly associated with epilepsy include infarction, intracerebral hemorrhage, aseptic meningitis, and psychosis. Although these studies have adjusted for some confounding factors, the nature of observational studies seems to make it impossible to entirely control for unmeasured risk factors. Furthermore, immunosuppressive medications such as cyclosporine and tacrolimus are associated with an increased risk of epileptic seizures, even though these medications are not frequently used in SLE at present.^24^ The above studies all agree that patients with SLE have a higher incidence of epilepsy. However, in our Mendelian randomization study, we did not find a genetic causal relationship between SLE and epilepsy.

Although this Mendelian randomization study did not find a direct causal relationship between SLE and epilepsy, it is important to recognize the potential role of individual SNPs and other genetic markers in the complex context of disease comorbidity. A bioinformatics study discovered that the ε4 variation of the apolipoprotein E (APOE) gene is associated with both temporal lobe epilepsy (TLE) and SLE. This finding underscores the importance of exploring other genetic hypotheses related to the comorbidity of SLE and epilepsy.^25^ APOE ε4 is a variant of APOE protein that is associated with SLE probably due to its disrupting effects on the oxidant defense and vascular functions.^26^ Previous studies have also found that APOE ε4 is associated with TLE through mechanisms involving amyloid deposition in the brain and impaired nerve damage repair mechanisms.^27^, ^28^, ^29^ Therefore, APOE ε4 may be associated with TLE and SLE through impairing lipid metabolism and vascular functions. These studies not only support the importance of exploring other genetic hypotheses related to the comorbidity of SLE and epilepsy but also provide a direction for future research, namely, investigating how these shared genetic variations affect the comorbidity of these diseases and their potential significance in developing new treatment strategies. These findings offer crucial biological insights into the complex relationships between these diseases and may guide future therapeutic research, thereby improving treatment outcomes for patients with these comorbid conditions.

In our research, no confounding SNPs related to the outcomes were identified using Phenoscanner. This finding suggests that the association between these specific genetic markers and epilepsy might be less significant than previously thought. However, we cannot deny the possibility of an epidemiological association between SLE and epilepsy. It is important to recognize that risk factors include age, income, central nervous system infections, and trauma, among others, which are crucial for early prevention of epilepsy.^30^, ^31^ Despite epidemiological evidence suggesting an association between SLE and epilepsy, several limitations should be considered when interpreting these findings.^22^ Firstly, it is essential to recognize that correlation does not imply causation, and establishing a causal relationship between SLE and epilepsy requires rigorous investigation beyond epidemiological observations. Secondly, information bias could affect the validity of the results, including recall bias or reporting bias, potentially leading to distorted conclusions. Additionally, the presence of confounding factors, such as shared genetic risk factors or environmental exposures, may complicate the interpretation of the relationship between SLE and epilepsy. Moreover, the potential for selection bias in epidemiological studies should be acknowledged, as the chosen study population may not fully represent the broader population. Finally, the inability to determine the temporal sequence of events is a significant limitation in epidemiological studies, as it remains unclear whether SLE precedes epilepsy or vice versa. It is also noteworthy that seizures do not equate to epilepsy. Some patients with lupus may experience acute symptomatic seizures due to lupus cerebritis, and these seizures will cease once the cerebritis is treated. Consequently, while epidemiological evidence suggests an association between SLE and epilepsy, these findings must be interpreted cautiously in light of the aforementioned limitations.

Our research has several limitations. First, we analyzed the causal relationship between SLE and only certain types of epilepsy. Second, the findings were based on European ancestry, and it remained uncertain whether they could be extended to other races. Further additional MR studies are required in different populations. While SLE has been explored as a potential risk factor for epilepsy, MR studies have not been previously performed. To our knowledge, this is the first such study on the causal relationship between SLE and epilepsy.

## 5 CONCLUSION AND FUTURE OF RESEARCH IN THE FIELD

In conclusion, the results of the Mendelian randomization analysis do not support a genetic causal relationship between SLE and an increased risk of epilepsy. However, further investigation is still necessary to elucidate the common pathophysiological mechanisms shared by these conditions. Future research should focus on the abnormal activities of the immune system and their impact on neurological functions, exploring the comorbidity patterns of epilepsy within SLE patients, and examining potential shared influencing factors, such as chronic inflammation, the role of autoantibodies, genetic predispositions, and environmental elements. Moreover, investigations into whether the use of immunosuppressants or other therapeutic drugs in SLE patients may alter the frequency and severity of epileptic seizures are imperative. Delving into these potential interactions will not only deepen our understanding of the relationship between SLE and epilepsy but also aid in the development of more personalized and effective treatment strategies, ultimately enhancing the quality of life for patients.

## AUTHOR CONTRIBUTIONS

Y Hu, Y Zhang, and G Li conceived and designed the study. Y Hu, D Lin, and D Wu conducted the formal analysis and developed the methodology. Y Hu wrote the initial drafts. D Wu and D Lin helped draft the manuscript. Y Zhang and G Li are the corresponding authors of this work and supervised work on the entire manuscript. All the authors read and approved the final manuscript.

## FUNDING INFORMATION

This research was funded by the Natural Science Foundation of China (NSFC, 82001434), the Natural Science Foundation of Chongqing (cstc2020jcyj‐msxmX0149 and CSTB2023NSCQ‐MSX0176), the China Postdoctoral Science Foundation (2022 M710562), and the Kuanren Talents Program of the second affiliated hospital of Chongqing Medical University (kryc‐yq‐2215).

## CONFLICT OF INTEREST STATEMENT

None of the authors has any conflict of interest to disclose related to this study. We confirm that we have read the Journal’s position on issues involved in ethical publication and affirm that this report is consistent with those guidelines.

## CONSENT FOR PUBLICATION

All authors agreed to the publication of this article.

## Supporting information


Figure S1.

Figure S2.

Table S3.

## Data Availability

The data that support the findings of this study are available from the corresponding author upon reasonable request.
