# Supplementary figures and images for: Systemic lupus erythematosus and epilepsy: A Mendelian randomization study
Source: Epilepsia Open. 2024 Sep 28;9(6):2274–82. doi: 10.1002/epi4.13058 (PMC11633673; doi:10.1002/epi4.13058)

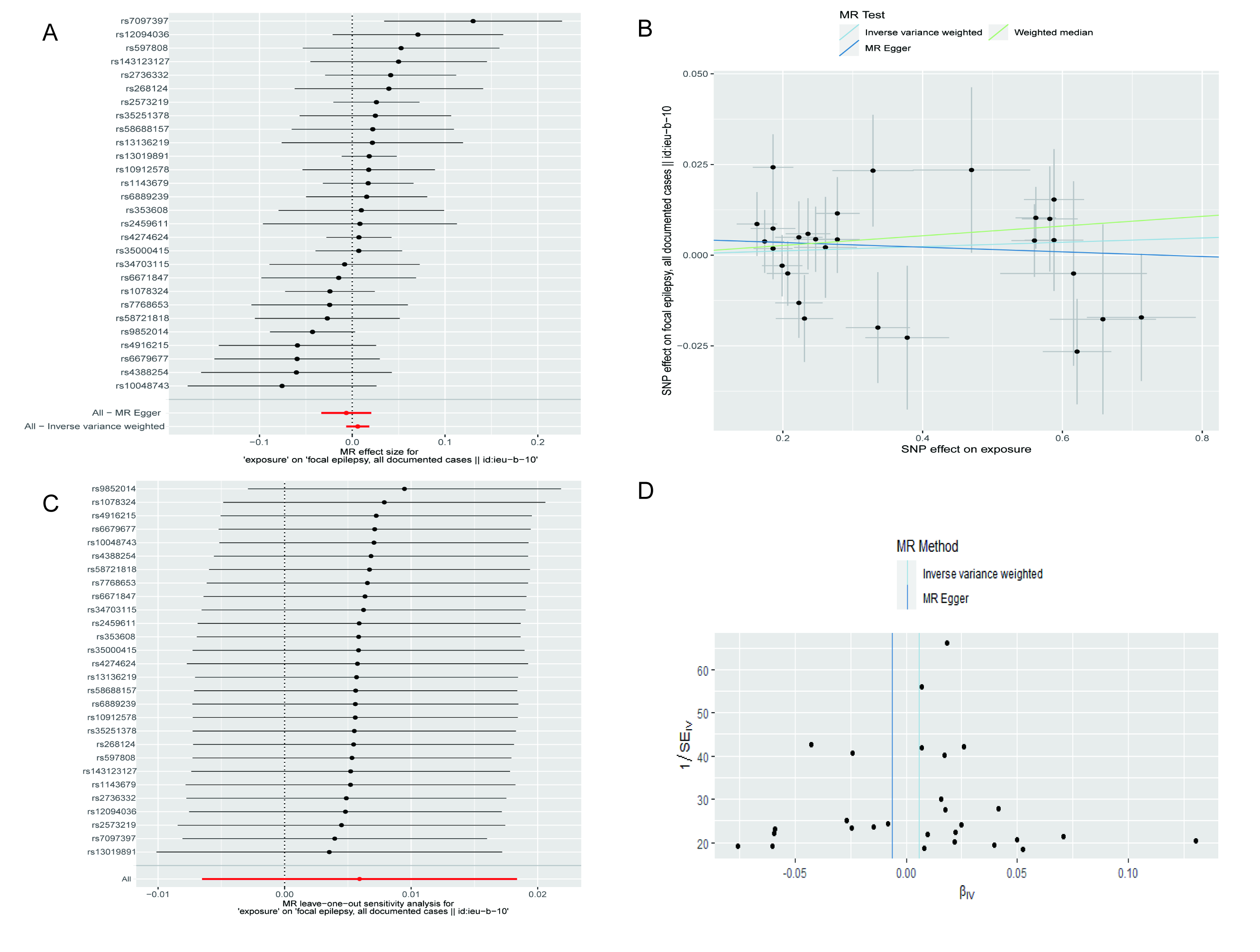

Supplement: Supplementary file 1 — Figure S1. [file EPI4-9-2274-s001.tif]

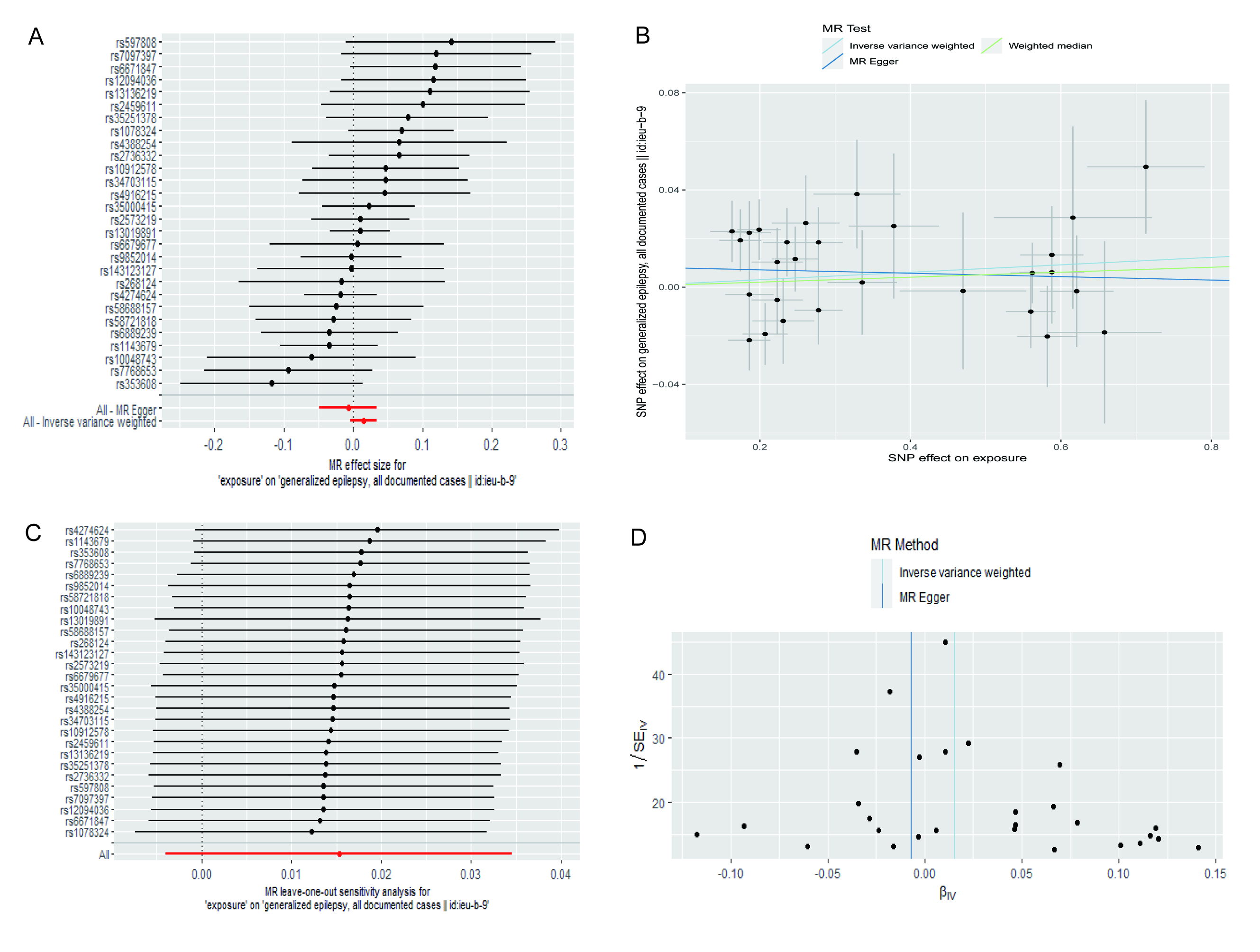

Supplement: Supplementary file 2 — Figure S2. [file EPI4-9-2274-s003.tif]
