# Supplementary material for: Systemic lupus erythematosus and epilepsy: A Mendelian randomization study
Source: Epilepsia Open. 2024 Sep 28;9(6):2274–82. doi: 10.1002/epi4.13058 (PMC11633673; doi:10.1002/epi4.13058)
Supplement: Supplementary file 3 — Table S3. [file EPI4-9-2274-s002.docx]

**Supplementary 3.** SLE SNPs and three type of epilepsy SNPs.

|  | SLE | All Epilepsy | Focal epilepsy | Generalised Epilepsy |
| --- | --- | --- | --- | --- |
| SNP | rs10048743 | rs35251378 | rs35251378 | rs58688157 |
| SNP | rs10200680 | rs9852014 | rs4388254 | rs10048743 |
| SNP | rs1078324 | rs1078324 | rs7768653 | rs9852014 |
| SNP | rs10912578 | rs10912578 | rs4274624 | rs4388254 |
| SNP | rs1143679 | rs4388254 | rs4916215 | rs6679677 |
| SNP | rs12094036 | rs7768653 | rs13019891 | rs10912578 |
| SNP | rs1270942 | rs35000415 | rs58721818 | rs4916215 |
| SNP | rs13019891 | rs6679677 | rs58688157 | rs1078324 |
| SNP | rs13136219 | rs12094036 | rs2459611 | rs35000415 |
| SNP | rs13332649 | rs268124 | rs7097397 | rs1143679 |
| SNP | rs143123127 | rs13019891 | rs10048743 | rs35251378 |
| SNP | rs143810596 | rs58721818 | rs597808 | rs13019891 |
| SNP | rs1464446 | rs597808 | rs1143679 | rs268124 |
| SNP | rs150180633 | rs7097397 | rs9852014 | rs13136219 |
| SNP | rs17849501 | rs2459611 | rs1078324 | rs6889239 |
| SNP | rs2431697 | rs4274624 | rs6889239 | rs2736332 |
| SNP | rs2459611 | rs13136219 | rs13136219 | rs7768653 |
| SNP | rs2573219 | rs58688157 | rs6679677 | rs58721818 |
| SNP | rs268124 | rs2736332 | rs12094036 | rs7097397 |
| SNP | rs2736332 | rs6889239 | rs353608 | rs353608 |
| SNP | rs34703115 | rs353608 | rs2736332 | rs597808 |
| SNP | rs35000415 | rs10048743 | rs10912578 | rs2459611 |
| SNP | rs35251378 | rs4916215 | rs35000415 | rs4274624 |
| SNP | rs353608 | rs1143679 | rs268124 | rs12094036 |
| SNP | rs3747093 | rs143123127 | rs143123127 | rs143123127 |
| SNP | rs4274624 | rs2573219 | rs2573219 | rs2573219 |
| SNP | rs4388254 | rs34703115 | rs34703115 | rs34703115 |
| SNP | rs4661543 | rs6671847 | rs6671847 | rs6671847 |
| SNP | rs4916215 |  |  |  |
| SNP | rs58688157 |  |  |  |
| SNP | rs58721818 |  |  |  |
| SNP | rs597808 |  |  |  |
| SNP | rs6671847 |  |  |  |
| SNP | rs6679677 |  |  |  |
| SNP | rs6889239 |  |  |  |
| SNP | rs7097397 |  |  |  |
| SNP | rs73050535 |  |  |  |
| SNP | rs73068668 |  |  |  |
| SNP | rs7768653 |  |  |  |
| SNP | rs7823055 |  |  |  |
| SNP | rs7899626 |  |  |  |
| SNP | rs9274357 |  |  |  |
| SNP | rs9852014 |  |  |  |
